# Home birth without skilled attendants despite millennium villages project intervention in Ghana: insight from a survey of women’s perceptions of skilled obstetric care

**DOI:** 10.1186/s12884-015-0674-1

**Published:** 2015-10-07

**Authors:** Emmanuel Kweku Nakua, Justice Thomas Sevugu, Veronica Millicent Dzomeku, Easmon Otupiri, Heather R. Lipkovich, Ellis Owusu-Dabo

**Affiliations:** School of Public Health, Department of Population, Family and Reproductive Health, Kwame Nkrumah University Science and Technology, PMB, Kumasi, Ghana; Henry Ford Health System, Department of Public Health Sciences, Detroit, MI USA; Department of Nursing, Faculty of Allied Health Sciences, Kwame Nkrumah University of Science and Technology, Kumasi, Ghana; Sekyere-Kumawu Health Directorate, Box 40, Kumasi, Ghana; School of Public Health, Department of International and Global Health, KNUST, Kumasi, Ghana; Kumasi Collaborative Center for Tropical Research, Kumasi, Ghana

**Keywords:** Maternal health, Rural, Millennium Villages Project, Skilled birth attendance

## Abstract

**Background:**

Skilled birth attendance from a trained health professional during labour and delivery can prevent up to 75 % of maternal deaths. However, in low- and middle-income rural communities, lack of basic medical infrastructure and limited number of skilled birth attendants are significant barriers to timely obstetric care. Through analysis of self-reported data, this study aimed to assess the effect of an intervention addressing barriers in access to skilled obstetric care and identified factors associated with the use of unskilled birth attendants during delivery in a rural district of Ghana.

**Methods:**

A cross-sectional survey was conducted from June to August 2012 in the Amansie West District of Ghana among women of reproductive age. Multi-stage, random, and population proportional techniques were used to sample 50 communities and 400 women for data collection. Weighted multivariate logistic regression analysis was used to identify factors associated with place of delivery.

**Results:**

A total of 391 mothers had attended an antenatal care clinic at least once for their most recent birth; 42.3 % of them had unskilled deliveries. Reasons reported for the use of unskilled birth attendants during delivery were: insults from health workers (23.5 %), unavailability of transport (21.9 %), and confidence in traditional birth attendants (17.9 %); only 7.4 % reported to have had sudden labour. Other factors associated with the use of unskilled birth attendants during delivery included: lack of partner involvement aOR = 0.03 (95 % CI; 0.01, 0.06), lack of birth preparedness aOR = 0.05 (95 % CI; 0.02, 0.13) and lack of knowledge of the benefits of skilled delivery aOR = 0.37 (95 % CI; 0.11, 1.20).

**Conclusions:**

This study demonstrated the importance of provider-client relationship and cultural sensitivity in the efforts to improve skilled obstetric care uptake among rural women in Ghana.

## Background

In low- and middle-income countries (LMICs), majority of births in rural areas occur outside a healthcare facility without skilled obstetric care [1, 2]. In rural areas of Ghana, 45 – 46.1 % [3, 4] of births are overseen by skilled birth attendants (a doctor, physician assistant, midwife or nurse). For the estimated 55 % of women who do not receive skilled attendance at birth, this care comes largely from traditional birth attendants (TBAs, 30 %) and relatives and friends (25 %) [3].

In contrast, more than 80 % of births by women in urban areas are supervised [4] by skilled birth attendants. Consequently, urban maternal mortality is markedly lower than that in rural communities [5]. Unskilled birth attendance is a major obstacle preventing the attainment of MDG 5 which seeks to reduce maternal mortality in LMICs [6]. About 75 % of rural maternal deaths are due to; haemorrhage, eclampsia, obstructed labour and puerperal sepsis as direct causes [6]. A substantial proportion of these deaths are preventable through appropriate and timely interventions, particularly improved access to health facility based intrapartum care [7, 8].

In Ghana, expectant mothers benefit from the National Health Insurance Scheme (NHIS) that covers the cost of all maternity services [9]. Insured women are more likely to seek medical care when ill than those not insured with the NHIS [10]. However, in most rural communities lack of skilled personnel in these health facilities and skilled obstetric care services negate the possible benefits of the NHIS. This often results in avoidable or otherwise preventable maternal death [11]. To combat this disparity, the Millennium Villages Project (MVP) designed a strategic model targeted at deprived communities to mitigate some of these inherent problems unique to rural areas in LMICs. The MVP sought to improve the living standards of rural communities through the coordinated and simultaneous delivery of proven package of interventions in health, agriculture, infrastructure and education [12].

This study aimed to assess the effect of an intervention by the MVP that was expected to address barriers to accessing skilled obstetric care and to identify factors associated with the use of unskilled birth attendants during delivery in a rural district of Ghana.

## Methods

### Setting

This cross sectional study, was carried out over the period June – August 2012, in the Amansie West District, a rural district located in the south-western part of the Ashanti Region of Ghana. Amansie West is one of the 30 administrative districts in the Ashanti Region, and one of the most deprived [13]. The district has seven (7) sub-districts with 162 communities. Amansie West district has only one health facility capable of offering comprehensive emergency obstetric care; this facility is far from the reach of many expectant mothers [14]. The district has 54 traditional birth attendants (TBAs) who conduct deliveries in the communities.

The study sites were communities in seven sub-districts including Keniago and Tontokrom, which received the intervention package from the Millennium Village’s Project (MVP) in 2006. A baseline assessment of skilled deliveries in these sub-districts was conducted prior to the inception of MVP with a reported, skilled delivery rate of 29 % [15]. The intervention package consisted of; construction of health centres, improved road infrastructure, and the provision and support of health staff (midwives, laboratory technologists, laboratory assistants, pharmacy technicians) to the health centres. In addition, MVP deployed health workers and health care assistants to the communities to provide free antenatal care […REF].

### Sampling and survey

A sample size of 400 mothers was estimated based on 40.9 % reported skilled deliveries in the district [14] with 5 % degree of error and 10 % non-response rate.

**Fig. 1:**
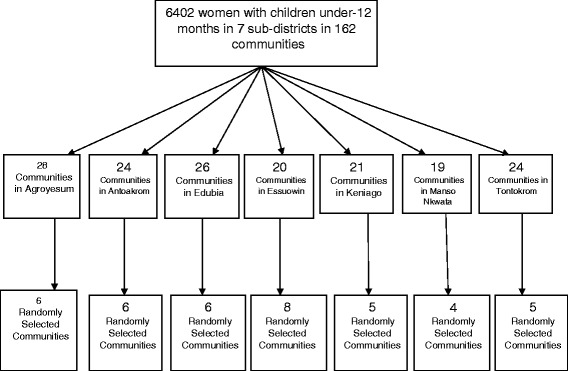
Study design and sampling

A multi-stage sampling method was used (Fig. 1). First, communities were selected by simple random sampling technique. The seven (7) sub-districts were then listed and 50 out of 162 communities in the district were randomly selected, out of which 15 were from the MVP intervention sub-districts. The second stage of selection involved sampling from a census list of local health officials-Community Health volunteers (CHW) and child welfare clinic personnel (CWC). Data obtained from the community health volunteers ensured that unskilled deliveries were captured. The two lists were put together and checked for consistency. The eligible study population was identified and a sampling frame (6,402 women attending post-partum care) from which mothers with children under-12 months were selected for inclusion from each community was prepared. Mothers aged 15–49 years in each community who had given birth in the year preceding the survey were eligible for inclusion in the study. Finally, the study participants were selected from the sampling frame from the 50 communities; we ensured representation from different age groups using a sampling proportional to size approach for each sub-district.

### Data collection

A structured questionnaire was used to collect data on participants’ socio-demographics, antenatal and postnatal care attendance. Also knowledge of the benefits of skilled delivery, and reasons for use or non-use of skilled birth attendants’ services during delivery were elicited. The structured questionnaire was pre-tested in Abuoboso, a community with similar characteristics as the study communities.

### Operational definitions

#### Birth preparedness and complication readiness (BPAR)

A pregnant woman is considered birth prepared if she and/or her family identifies a skilled birth attendant; identifies the location of the closest appropriate healthcare facility; has funds for birth-related and emergency expenses; has transport to the health facility for the birth and obstetric emergency, and has identified compatible blood donors in case of emergency [16].

Skilled birth attendant: persons with midwifery skills (doctor, nurse, midwives and health officer) who can manage normal deliveries and diagnose, manage or refer obstetric complications.

### Ethical consideration

The Committee of Human Research, Publications and Ethics of the Kwame Nkrumah University of Science and Technology-School of Medical Sciences and Komfo Anokye Teaching Hospital, Kumasi, Ghana provided ethical approval for the study. Permission was obtained from the Amansie West District Health Directorate prior to the survey. Written informed or thumb print as well as verbal consent was obtained from all participants and no personal identifiers were collected.

### Data handling and statistical analysis

Filled questionnaires were checked immediately for completeness and accuracy for each respondent survey for completeness. Data were entered into Microsoft Access (Redmond, WA, USA) and exported into STATA version 12 (College Station, TX, USA) for cleaning and analysis. Data were analyzed using survey-sampling weights. Each sub-district total population size was taken from the Ghana 2010 Housing and Population Census. Population level weights (W_pi_) and women in communities with children under 12 months weight (W_wci_) were estimated for each community. The overall weight (W_TOTi_) was obtained by multiplying W_pi_ and W_pci_. The knowledge level of participants on the risk of unskilled delivery was assessed using a Likert scale and described using the median value. Poor knowledge was considered below the median and high knowledge above the median.

Proportions were presented for categorical variables and their associations determined by Chi-square test. A logistic regression model was fitted to estimate independent associations between unskilled delivery and predictor variables that were independently significant (*p* ≤ 0.05) in the univariate analysis. In the subsequent steps, variables that were not predictors were entered into the final model one at a time and retained as multivariate predictors using the Hosmer-Lemeshow goodness-of-fit test. A backward stepwise analysis was performed containing all the variables to identify the variables that were removed from the model. The most non-significant variables were considered first for removal. A goodness-of-fit test using Hosmer-Lemeshow test was conducted and found that the final model was appropriate (*p* = 0.99).

## Results

The majority of the participants (73.6 %) were between 20 and 34 years, 90.9 % were married and 46.6 % lived within 5 km of a health care facility. The main occupation of many participants (51.4 %) were subsistence farming and 65.2 % earned an average household income less than US$1 per day (Table 1).

**Table 1:** Socio-demographic and socioeconomic characteristics of study sample

Characteristics	Weighted			
	Non-MVP (*n* = 29163) %^a^	MVP (*n* = 11341) %^a^	Overall (*n* = 40504) %^a^	*P*-value
Age (Years)				0.36
15-19	12.3	10.5	11.8	
20-34	71.7	78.5	73.6	
35-49	16.0	10.9	14.6	
Marital status				0.01
Single**	7.2	15.7	9.4	
Married/Cohabitating	92.7	84.9	90.9	
Education level				0.56
None	22.3	20.2	21.8	
Primary	19.3	26.8	21.4	
MS/JHS	52.2	45.9	50.4	
SHS/above	6.2	7.1	6.5	
Occupation				
Farmer	50.8	52.9	51.4	0.93
Trading (small scale)	26.5	25.6	26.3	
Galamsey	22.7	21.5	22.4	
Number of children alive				0.41
1-2	48.7	40.9	46.6	
3-4	28.7	32.8	29.8	
5+	22.6	26.3	23.7	
Social economic atatus				0.68
Low	53.4	55.9	54.1	
High	46.6	44.1	45.9	
Distance to health facility				0.47
0-5	45.9	48.3	46.6	
6-10	37.4	40.0	38.1	
11+	16.7	11.7	15.3	
Average income of mother per day				0.001
<2 Ghana Cedis ($1)	60.0	78.7	65.2	
2-10 Ghana Cedis ($2-$5)	26.8	20.4	25.0	
>10 Ghana Cedis (>$10)	13.2	0.9	9.8	

^a^The % shown is percentage of n; **Single includes divorced, separated and widow

From Table 2, (97.7 %) of the participants attended an antenatal care clinic (ANC) at least once, 75.1 % had a minimum of 4 visits and most of the ANC services were provided by a midwife (91.4 %). Despite high ANC attendance, participants reported poor knowledge of risks associated with pregnancy (44.5 %). Although most participants reported knowing the benefits of skilled delivery (83.6 %), 54.8 % had ever delivered at least one of their children without a skilled care, and 42.3 % reported delivering their most recent child without skilled care.

**Table 2:** Reproductive characteristics of mothers in Amansie West District, Ghana

Characteristics	Unweighted		Weighted		
	Per cent %	Frequency *n* = 400	Per cent %	Frequency (*n* = 40504)	95 % CI
Received ante-natal care					
Yes	97.8	391	97.7	39583	96.2 to 99.2
No	2.2	9	2.3	921	0.7 to 3.8
ANC provider					
Doctor	2.8	11	2.8	1121	1.2 to 4.4
Midwife	91.0	364	91.4	37023	88.8 to 94.0
Auxiliary midwife/nurse	4.5	18	4.0	1602	2.2 to 5.7
Relatives/Friends/Self	1.8	7	1.8	758	0.4 to 3.3
Number of ANC visits					
<4 Visits	23.5	94	23.0	9118	18.9 to 27.2
4+ visits	72.6	291	75.1	29724	70.9 to 79.3
Don’t know	3.9	15	1.9	741	0.7 to 3.2
Birth preparedness^a^					
Yes	60.8	242	62.4	25283	57.8 to 67.1
No	39.2	158	37.6	15221	32.9 to 42.2
Knowledge of benefits of skilled delivery					
Yes	83.5	334	83.6	33853	79.9 to 87.2
No	16.5	66	16.4	6651	12.7 to 20.1
Knowledge level of unskilled delivery					
Poor knowledge	44.8	179	44.5	18029	39.6 to 49.4
Good knowledge	55.2	221	55.5	22475	50.6 to 60.3
Partner involved in decision making					
Yes	50.4	207	52.1	21098	47.1 to 57.1
No	49.6	193	47.9	19406	42.9 to 52.9
Ever delivered at home					
Yes	54.3	217	54.8	22198	49.8 to 59.8
No	45.8	183	45.2	18306	40.2 to 50.2
Place of delivery of last birth					
Unskilled home delivery	43.3	173	42.3	17126	37.4 to 47.2
Health facility skilled delivery	56.7	227	57.7	23378	52.8 to 62.6

^a^Birth preparedness, women expected to identify five basic actions they did before delivery: 1. identified a skilled birth attendant, 2. identify the location of the closest appropriate care facility, 3. raising funds for birth-related and emergency expenses, 4. arrangement for transport to a health facility for the birth and obstetric emergency and 5. Identification of compatible blood donors in case of emergency

**Fig. 2:**
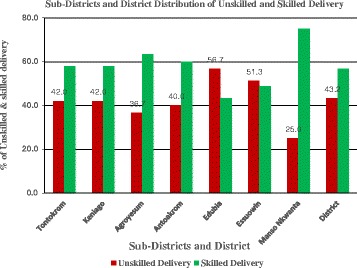
Proportion of unskilled and skilled deliveries by sub-district at Amansie West, Ghana

Figure 2 shows the proportion of unskilled and skilled deliveries. An average of 43.2 % unskilled deliveries occurred without a skilled birth attendant in the district and 25.0 % to 57.0 % in the sub-districts. Each of the two intervention sub-districts, Keniago and Tontokrom had 42.0 % deliveries while Edubia (non-intervention group) had the highest unskilled deliveries (56.7 %). Edubia has a high number of trained TBAs in the district who are perceived by expectant mothers to be “good” and whose practices may be culturally sensitive and consistent with the belief system of the rural woman while Essuowin (51.3 % unskilled deliveries) had inadequate health facilities in this sub-district compared with the others and this may have influenced more births with an unskilled birth attendant in the area. Manso Nkwanta has experienced a high influx of people for small-scale mineral mining and coupled with a common practice of health workers to remind women to report to deliver without ANC history may have influenced the large gap between unskilled deliveries and institutional deliveries in Manso Nkwanta sub-district.

The main reasons for participants choosing unskilled delivery over a health facility with skilled delivery were ‘insults from health staff’ (23.5 %), lack of transportation (21.9 %) and the availability of trained . traditional birth attendants and thus there was no reason to travel to access skilled delivery (17 %). Only 4.4 % of women cited lack of money as a primary reason for not attending a health facility for birthing (Table 3).

**Table 3:** Primary reasons stated for home delivery

Reasons	Unweighted		Weighted	
	Per cent %	Frequency (*n* = 173)	Per cent %	Frequency (*n* = 17126)
Insults	23.7	41	23.5	4023
No transport	21.4	37	21.9	3756
TBA is good	17.3	30	17.9	3073
Cultural beliefs	13.9	24	13.5	2318
Sudden labour	6.9	12	7.4	1264
Delay	5.8	10	5.9	1015
Poor service	5.2	9	4.1	700
No money	4.6	8	4.4	753
Long distance	1.2	2	1.3	225

Table 4 reports factors associated with place of delivery in a rural setting. In the multivariate analysis, women in the MVP intervention sub-districts were less likely to deliver with a skilled birth attendant or at a health facility when compared with women from other districts. Factors associated with utilization of skilled delivery care were having never had an unskilled delivery, and attending four (4) or more ANC sessions. In contrast, those who lived far away from a facility (aOR = 0.32; 95 % CI 0.13 - 0.74), lacked partner involvement (aOR = 0.03; 95 % CI 0.01-0.06), and lacked knowledge of the benefits of skilled delivery (aOR = 0.37; 95 % CI 0.11-1.20) were less likely to delivery at a health facility.

**Table 4:** Factors associated with place of delivery in a rural setting^a^

Characteristics	Unadjusted OR(95 % CI)	Adjusted OR(95 % CI)
Intervention		
Non-MVP sub-districts	1.00	1.00
MVP sub-districts	1.02(0.63, 1.62)	0.48(0.19, 1.22)
Socio-demographic and socio-economic factors		
Age group		
15-19	0.87(0.47, 1.62)	0.47(0.15, 1.49)
20-34	1.00	1.00
35-49	1.12(0.63, 2.01)	3.12(0.91, 10.65)
Marital status		
Married	1.00	1.00
Single	0.7(0.36, 1.38)	3.32(0.91, 12.03)
Education Level		
None	1.00	
Primary	1.01(0.54, 1.87)	
Middle/Junior high School	1.73(1.02, 2.92)	
SHS/above	1.59(0.63, 4.00)	
Occupation		
Farmer	1.00	
Business	1.98(1.13, 3.06)	
Galamsey	1.52(0.91, 2.54)	
Number of children		
1-2	1.00	
3-4	0.73(0.45, 1.16)	
5-6	0.80(0.48, 1.33)	
Average household income per day		
$1	1.00	
$2-$5	1.48(0.91, 2.42)	
$5	2.49(1.15, 5.41)	
Client factors:		
Distance		
≤5	1.00	1.00
6-10	0.45(0.29, 0.71)	0.32(0.13, 0.74)
11-15	0.52(0.28, 0.97)	0.40(0.11, 1.46)
Birth preparedness		
Yes	1.00	1.00
No	0.05(0.03, 0.09)	0.05(0.02, 0.13)
Partner involvement in decision making		
Involved	1.00	1.00
Not involved	0.02(0.01, 0.04)	0.03(0.01, 0.06)
Ever had unskilled delivery		
Yes	1.00	1.00
No	5.19(3.31, 8.13)	6.55(2.47, 17.34)
Knowledge of benefits of skilled delivery		
Yes	1.00	1.00
No	0.07(0.03, 0.15)	0.37(0.11, 1.20)
Number of ANC visits		
<4	1.00	1.00
≥4	3.32(2.04, 5.41)	1.36(0.51, 3.62)

^a^Table 4 estimates weighted for sampling probability

## Discussion

This study aimed to assess the effect of the MVP on the uptake of skilled delivery care and to identify factors associated with unskilled delivery in a rural district in Ghana. In the 5 years between implementation of the MVP intervention package and subsequent survey, a notable uptake of skilled delivery has been observed. Across the district, unskilled deliveries decreased by 27.8 % across the district. Even though Keniago and Tontokrom were sub-districts that had the intervention package, unskilled deliveries reduced by only 23.0 %. There still remained the common practice of not accessing the services of skilled attendants in spite of the availability of the services. However, Amansie West district as a whole has experienced a decline in unskilled delivery.

### Factors that enhance the use of skilled delivery services

The continuous use of unskilled deliveries in the MVP intervention communities suggests that merely making quality obstetric care services available is not enough to influence higher uptake of supervised delivery services, similar to previous reports in rural areas in LMIC [17–19]. Despite the high ANC visits by expectant mothers in this study, acceptability to use health facilities during deliveries is low in the rural communities in LMICs [20]. Affordability and availability were previously reported [6, 21, 22] to increase access to skilled birth attendants in rural LMIC. In this study, we reported only 4.4 % of mothers who could not access skilled delivery due to financial difficulties. It is therefore imperative to understand why many expectant mothers would still choose to deliver without skilled care in spite of the very high chance of maternal complications and/or death. The success or failure of any public health program depends largely on the public’s effective use of the services offered. Majority of the participants had only basic or no education and may not totally appreciate the benefits of skilled delivery at a health facility. Many human factors can also thwart access and use of curative medications in resource-poor settings, including traditional beliefs, illiteracy, and fatalistic attitudes. We observed that many participants’ knowledge regarding benefits of skilled delivery and the risks involved in unskilled delivery was rather poor.

Some of the main reasons reported by mothers for choosing unskilled delivery over a skilled facility were; poor attitude and verbal abuse from the skilled service providers, transportation challenges, and the availability of a trained traditional birth attendant in their community. Negative previous experiences from service providers might have deterred some of the women from using skilled birth attendants [23, 24].

Training of staff on customer care and cross-culture communication can be used to enhance effective communication between health workers and clients to remove the latter’s fear of unpleasant attitude of the former. Mwifadhi et al. [23] found lack of money, sudden onset of labour, tradition and culture as determinants for the place of delivery. The presence of a network of trained TBAs in the communities has given an alternative source of services to a health facility with little or no access cost. It is obvious that persistent health education is crucial to ensure effective use of the services offered, especially to those at risk. MVP intervention might not have considered health education as a tool to the deployment of the intervention. One other way to reducing unskilled deliveries substantially in rural settings is to identify, train and incentivize TBAs to refer all women requiring obstetric services to the nearest health facility. This can be effective and sustainable if an incentive package is tied to the number of cases TBAs refer to a health facility to guarantee their livelihood. This is consistent with other studies in LMICs that reported the strong position of TBAs in communities in which they operate as the preferred service provider because of their cultural sensitivity, easy availability and cheaper service [24, 25]. Cost and distance to the health facility were the two least reported problems by mothers in Amansie West District.

Despite the fact that most of the women were very close to skilled care (<5 km), nearly half of the women used unskilled delivery services. This indicates that some pre-hospital care services, local improvisations should be considered.

### Effect of Millenium Village Project (MVP) Intervention

Five years into the intervention, the two intervention sub-districts Keniago and Tontokrom were still recording high (42.0 %) deliveries outside health facilities, similar to the overall district average of 43.2 %. It was hypothesized that the MVP intervention, in the two sub-districts would result in a greater proportion of women utilizing healthcare facility for delivery compared with the other sub-districts in the District [12]. However, only two non-intervention sub-districts, Edubia and Essuowin, had higher unskilled deliveries than the two intervention sub-districts. The other three non-intervention sub-districts that recorded lower unskilled deliveries may have some inherent advantages that the rest of the sub-districts did not have. These could include better maternal health seeking behavior, higher income levels, higher education status and healthy provider-client relationship among others. The two sub-districts of Keniago and Tontokrom, may have peculiar characteristics and obstacles other than those mitigated by the intervention package and which were not accounted for by the intervention. No single intervention therefore, is appropriate in all contexts. Interventions should be adapted to specific local ecological, epidemiological, economic, and social conditions. Campbell et al. [26] stated that there are many single interventions available, but none alone can effectively achieve the high coverage expected [27]. It is in this regards that one had to evaluate the adequacy of interventions put in place to enhance utilization of healthcare facilities for delivery.

### Maternal mortality ratio with the introduction of MVP

As anticipated, maternal mortality ratio in the entire Amansie West District decreased in accordance with the decrease in percentage of unskilled births between 2007 and 2011, except for 2010 when unskilled delivery rates declined by 11.8 percentage points from the preceding year while the maternal mortality increased by 5 deaths per 100,000 births in the same time frame. While this overall drop in the maternal mortality ratio is certainly encouraging, it does not give enough insight into how maternal mortality ratios in Keniago and Tontokrom were influenced by the use of skilled delivery services or how they influenced the overall trend of decreasing maternal mortality in Amansie West [14].

In this study nearly all the women had at least one ANC visit with majority of them making either four or more visits. This however did not translate into high skilled delivery as reported by similar studies [28, 29]. High ANC visits by the women do present opportunity for health education on risks and complications for both the mother and the unborn child during the pregnancy [25]. Single mothers and older women (35–49 years) were three times more likely to use unskilled services compared with married and younger women. Women who had ever used unskilled attendants during delivery were six times more likely to have an unskilled delivery than those who used skilled delivery services in a health facility. Women who prepared resources for birth were more likely to deliver at a health facility. Hence, birth preparedness is a preventive behavior [26, 28, 30–32] implying that adequate knowledge on pregnancy and delivery could strongly influence uptake of skilled delivery [29]. Partner involvement in decision making influenced place of delivery and this finding was consistent with studies in Ethiopia, Kenya and Vietnam [33, 34].

### Strengths and limitations

This study provides data on factors influencing the choice of place of delivery in rural Ghana. In particular, it provides contextual factors influencing the utilization of skilled obstetric care which can inform policy programming and appropriate intervention. We were however unable to draw direct attribution of institutional factors, content and quality of antenatal and delivery care benefit to the outcome of this evaluation in a cross sectional study. The sample of women seeking post-partum care excluded other women who may have different characteristics from those attending the clinics. These do not render the study results invalid.

## Conclusion

The significant investment and intervention package made by MVP to reduce the barriers to accessing skilled obstetric care and delivery, may have contributed to an improvement in the uptake of skilled delivery after five years of intervention across the district. However some sub-districts did not show marked improvements. Maternal factors such as age, education, marital status, occupation, ever used unskilled delivery services, number of ANC visits, partner support and the services of TBA in the communities were more likely to influence choice of place of delivery. Additionally, the lack of knowledge of the benefits of skilled delivery and, verbal abuse and poor attitude experienced from skilled birth attendants deterred expectant mothers from accessing skilled delivery. The success of this health intervention would depend largely on the effective public education and communication among health workers and clients.

## Abbreviation

LMICs: Low-and middle income countries
TBAs: Traditional birth attendants
MDG: Millenium Development Goal
NHIS: National Health Insurance Scheme
MVP: Millenium villages project
ANC: Antenatal care
MMR: Maternal mortality ratio
CWCs: Child welfare clinic
